# Socio-Demographic Characteristics, Dietary, and Nutritional Intakes of French Elderly Community Dwellers According to Their Dairy Product Consumption: Data from the Three-City Cohort

**DOI:** 10.3390/nu12113418

**Published:** 2020-11-07

**Authors:** Hermine Pellay, Corinne Marmonier, Cécilia Samieri, Catherine Féart

**Affiliations:** 1 INSERM, University Bordeaux, BPH, U1219, F-33000 Bordeaux, France; hermine.pellay@u-bordeaux.fr (H.P.); cecilia.samieri@u-bordeaux.fr (C.S.); 2 CNIEL, Service Recherche Nutrition-Santé, F-75009 Paris, France; cmarmonier@cniel.com

**Keywords:** dairy products, energy intake, food intakes, nutrient intakes, aging, population-based cohort

## Abstract

Few data are available regarding dietary habits of the elderly, especially about dairy products (DPs) (total DP and milk, fresh DP, and cheese), whereas these are part of healthy habits. The aim was to describe the socio-demographic characteristics, food, and nutritional intakes of elderly DP consumers. The sample consisted of 1584 participants from the Three-City-Bordeaux cohort (France), who answered a food frequency questionnaire and a 24-h dietary recall. Socio-demographic characteristics, practice of physical activity, Body Mass Index, and polymedication were registered. The sample was 76.2 years (SD 5.0 years) on average, 35% were in line with the French dietary guidelines for DP (3 or 4 servings of DP/day), while 49% were below, and 16% above. Women were significantly more likely to declare the highest total DP (≥4 times/day), milk (>1 time/day), and fresh DP (>1.5 times/day) frequency consumption. The highest cheese frequency consumers (>1.5 times/day) were more likely men, married, and ex-smokers. The highest frequency of fresh DP intake was significantly associated with the lowest energy and lipid intakes, and that of cheese with the highest consumption of charcuteries, meat, and alcohol. This cross-sectional analysis confirmed that the socio-demographics and dietary characteristics varied across DP sub-types consumed, which encourages individual consideration of these confounders in further analyses.

## 1. Introduction

Longevity has remarkably increased over the past decades, notably in developed countries. In France, healthy life expectancy was 63.9 years on average in 2018 and life expectancy at birth is expected to increase by 5 years between 2018 and 2050 for both genders. Moreover, it is estimated that more than one person out of four will be 65 years old in 2050 [1]. This increased proportion of older adults will result in increasing demands of healthcare and medical services. Therefore, maintaining healthy aging represents a tremendous social and economic challenge across the world [2].

Eating a well-balanced diet coupled with regular physical activity are well-known lifestyle factors to promote health; this holds to all age groups but is specifically crucial for healthy aging, which depends on lowering the risk of non-communicable diseases and on maintaining physical and mental capacities in the elderly [3]. Because of age-related physical, physiological, and psychosocial changes, meeting the nutritional needs of older adults through diet can be challenging. Dietary guidelines recommend a well-balanced diet including major food groups for appropriate intake of essential macro- and micro-nutrients [4,5]. As several nutrients (including vitamins D, B1, and B2; calcium; magnesium; and selenium) have been identified at risk of inadequate intake among older adults, it suggests that attention should be paid to the consumption of their main providers [6]. Therefore, dairy products (DPs), which provide proteins of high quality, and numerous nutrients, vitamins, and minerals [6,7,8,9], are part of most food-based dietary guidelines that promote a healthy diet [10,11,12]. Note that DP as a whole are a heterogeneous food group, which encompass milk, fresh DP (yogurt/cottage cheese/petit suisse), and cheese, and their nutrient contents vary according to the sub-type [13] and that lactose intolerance or allergies might reduce their consumption.

Regarding health, a higher DP consumption has been associated with several age-related benefits, such as a lower risk of death, hypertension, type 2 diabetes, and metabolic syndrome and improved bone health [14,15,16,17,18,19]. The type of DP appears as a key component of such associations [20,21]. For instance, in a meta-analysis on 938,415 participants and 93,518 mortality cases, Guo et al. reported a lack of association between total dairy (high- or low-fat) and milk with the risk of death, while an inverse association between total fermented dairy (including sour milk products, yogurt, or cheese; +20 g/day) and a significant 2% reduced risk of all-cause mortality and cardiovascular diseases [22]. Moreover, the foods consumed in combination with DP (i.e., the food matrix) [23], the dairy structure, and the SFA contents of these DPs appear also as key factors of potential DP-related health outcomes [24]. Although the DP fats content is mostly saturated (65%), it does not seem to adversely affect cardiovascular risk, while debate still remains regarding the SFA recommendations that should be applied, particularly among older adults [5,25,26,27,28].

To our knowledge, few studies so far have assessed the contribution of DP consumption on nutritional status (limited to vitamin and nutrient status) in older adults; these few existing studies have highlighted that DP consumption significantly contributed to the protein, SFA, B-, and D vitamins status depending on the DP sub-type among this vulnerable population [29,30]. No study has yet characterized, as a whole, the sociodemographic criteria, dietary patterns (i.e., describing the food group intakes), and nutrient intakes of elderly dairy consumers. Several reports have nevertheless pointed out the need for carefully considered gender, socio-demographic, socio-economic status, and lifestyle characteristics, which might improve the efficiency of targeted public health messages among the oldest old [31,32,33]. Therefore, the present study aimed to describe the socio-demographic characteristics, dietary habits, and nutrient intakes according to the frequency of consumption of total DP and DP sub-types of French older adults.

## 2. Methods

### 2.1. Study Overview

The Three-City Study (3C) is an ongoing population-based study conducted in three French cities (Bordeaux, Dijon, Montpellier, France). This cohort was initiated in 1999–2000 to study the vascular risk factors of dementia [34]. Its protocol was approved by the Consultative Committee for the Protection of Persons participating in Biomedical Research at Kremlin-Bicêtre and all participants gave written informed consent. Participants were randomly sampled from electoral rolls. To be eligible, participants had to be 65 years and older at the time of recruitment and not institutionalized. Among the 9294 participants, 2104 were from the Bordeaux center where the initial data collection was completed in 2001–2002 (wave 1) with a comprehensive dietary survey among 1755 participants.

### 2.2. Assessment of Food and Nutrient Intakes

#### 2.2.1. Dairy Products

A team of trained dieticians visited all participants at home between 2001 and 2002. Two types of dietary surveys were administered during face-to-face interviews to assess dietary habits. First, a Food Frequency Questionnaire (FFQ) allowed assessment of the daily frequency consumption of 148 foods and beverages (with frequencies assessed in 11 classes, from “never or less than once a month” to “7 times per week”) during each of the six meals/snacks of the day, as previously detailed [35]. Regarding DP, the following items were considered: consumption of “coffee with milk”, “tea with milk”, “chocolate”, “chicory”, “natural milk or with cereal”, and “milk” were considered by adding each response in a single variable called “milk”; those of “yogurt and cottage cheese” were considered as the “fresh DP category” while those of “cheese” were classified as the “cheese” category.

In addition to the FFQ, a 24-h dietary recall was administered at home [36]. Briefly, it allowed estimation of the total amount of all foods and beverages spontaneously ingested the day before the interview, and during and between meals; the 24-h recall was complementary to the FFQ, as it provided greater detail in the food items evaluated along with the quantities consumed daily. No weekend day was recorded. Photographs were used to precisely assess quantities [36]. Therefore, the total amount of DP and of each DP sub-type can account for servings (i.e., amount) and then be compared with the French recommended dietary allowances (RDAs) applyied in 2001 and still in progress today [4].

Using the 24-h dietary recall, 673 foods and beverages were spontaneously reported and we identified 7 items that could be attributed to the “milk” category (expressed in mL); 19 items that could be attributed to the “fresh DP category”, including cottage cheese and petit-suisse (expressed in g); and 47 items that could be attributed to the “cheese” category (expressed in g). For each DP subclass, a typical serving was defined as follows: 150 mL of milk (category of milk); 15 g of concentrated milk/skimmed and semi skimmed milk powder (category of milk); 18 g of whole milk powder (category of milk); 125 g of yogurt (category of fresh DP); 100 g of cottage cheese/petit-suisse (category of fresh DP); and 30 g of cheese.

Data about food intakes from both dietary surveys were significantly correlated in an independent sub-sample of the 3C study [37].

#### 2.2.2. Other Food Groups Intake

From the FFQ, we also considered the daily frequency consumption of 19 predetermined food groups, as follows: cereals/bread, pulses, pasta, potatoes, rice, biscuits/cakes, sweets/chocolate/soda, pizza/sandwich, raw vegetables/salad, cooked vegetables, fruits, charcuterie, fish/seafood, eggs, meat, poultry, coffee, tea, and alcohol [35]. As for DPs, all items were again recorded in 11 classes for each of the 3 main meals and 3 between-meal snacks.

#### 2.2.3. Energy and Nutrient Intakes

From the 24-h dietary recall, as previously described, we used the BILNUT^®^ software (SCDA Nutrisoft, Cerelles, France) to determine the total daily energy intake (without considering the energy provided by the alcohol intake), the daily macronutrients intake (i.e., carbohydrates, fatty acids (SFA, mono-unsaturated fatty acids (MUFAs) and polyunsaturated fatty acids (omega-3 and omega-6 PUFAs), proteins (from animal and vegetable origins)) and the daily micronutrients intake (including those relevant to the DP intake) [36]. We also identified participants consuming ≥1 g of proteins/kg of body weight/day and those consuming ≥1200 mg of calcium per day as participants in line with the current RDA for older adults, respectively [11,38,39].

### 2.3. Socio-Demographic and Lifestyle Characteristics

From the 3C database, we retained the following socio-demographic and lifestyle data: sex; age; education (in three categories: no education or primary school, secondary or high school, university); marital status (in four classes: married; divorced or separated; widowed; single); monthly income (in five classes: very low (less than 750€); low (750€ to 1500€); average (1500€ to 2250€); high (more than 2250€); refused to answer, including those who did not know their monthly income); polymedication, as the number of drugs/day ≥ 6; social isolation, combining living alone and feeling lonely “often enough” or “frequently”; smoking status (in three classes: never smoker; ex-smoker; current smoker); stoutness according to measured BMI and using the most relevant thresholds for identifying malnutrition among older adults [40] (in three classes: thinness (if BMI < 20 kg/m^2^ and age < 70 years) OR (if BMI < 22 kg/m^2^ and age ≥ 70 years); normal (if BMI (20–27) kg/m^2^ and age < 70 years) OR (if BMI (22–27) kg/m^2^ and age ≥ 70 years); overweight/obesity if BMI > 27 kg/m^2^); and practice of physical activity (in three classes: yes, no, no answer) [36,39].

### 2.4. Statistical Analyses

The SAS statistical software program (version 9.3; SAS Institute Inc., Cary, NC, USA) was used for statistical analyses.

We chose to divide the studied sample according to the usual frequency of consumption of (i) total DPs and (ii) milk, fresh DPs, and cheese, both evaluated by the FFQ: 3 categories per DP intakes were built, based on the quartile distribution of consumptions (low frequency: first quartile; moderate frequency: quartiles 2 and 3; high frequency: fourth quartile). This categorization ensured the identification of the most infrequent and frequent consumers. The FFQ database was preferred to define the main exposure, since a single 24-h dietary recall was available.

Then, socio-demographic characteristics, lifestyle, and dietary data (i.e., mean daily energy, macro- and micro-nutrient intakes from the 24-h recall, DPs, and all other food group consumptions from the FFQ) were described according to the 3 categories of frequency of consumption of total DPs and of DP subtypes.

Chi-Squared and ANOVA tests were used as appropriate. The Tukey–Kramer post hoc test was used to compare each mean between them (if ANOVA provided significant results). Statistical significance of different tests was accepted at *p*-value < 0.05.

## 3. Results

Among 1755 participants enrolled in the 3C Bordeaux cohort and followed at wave 1, 1606 answered the FFQ and 1658 answered the 24-h dietary recall, leading to a studied sample of 1584 participants with no missing data on the main exposure (i.e., total DP, milk, fresh DP, and cheese consumption) for the present analysis. The studied sample was 76.2 years old (SD 5.0 years) on average (ranging from 67.7 to 94.9 years), and 62.0% were women.

### 3.1. Total Dairy Products

Based on FFQ data, we stratified the sample as low daily frequency consumers of total DPs, such as those who consumed ≤ 2 times DPs per day (*n* = 394, 24.9% of the sample), moderate consumers who consumed 2–4 times DPs per day (*n* = 820, 51.8%), and high consumers who consumed ≥ 4 times DP per day (*n* = 370, 23.3%) (Table 1). Regarding the socio-demographic characteristics and lifestyle data, participants with the highest daily DP frequency intake were significantly more likely to be women (68.1% for the highest DP intake tertile, 56.6% for the lowest one), never smokers (68.4% for the highest DP intake tertile, 53.0% for the lowest one), and less often physically inactive (49.7% for the highest DP intake tertile, 59.7% for the lowest one) (Table 1).

Participants who declared the highest daily DP frequency intake also significantly reported a higher total energy intake (around +200 kcal/day for the highest DP intake tertile compared with the lowest one), and higher consumption of all macronutrients (including SFAs among total fatty acids and proteins from animal sources among total proteins) compared with others. Consistently, we observed that micronutrient intakes, such as calcium, phosphorus, zinc, and vitamins B1, B2, and B12, were significantly higher among participants with the highest frequency consumption of total DP, compared with others (Table 2).

In the study of all food groups recorded in the FFQ database, when the frequency of consumption of total DP was highest, the frequency of consumption of biscuits, sweets, and cooked vegetables was highest, while the frequency of consumption of charcuterie, meat, coffee, and alcohol was lowest (Table 3).

The consumed amounts of milk, fresh DPs, and cheese were significantly higher when the daily frequency consumption of total DP was the highest (Table 4). Participants with the highest frequency of total DP per day consumed 187 mL (SD 185 mL) of milk, 123 g (SD 111 g) of fresh DP, and 53 g (SD 45 g) of cheese per day on average.

### 3.2. Sub-Type of Dairy Products Consumed (Milk, Fresh DP, Cheese)

Based on the FFQ data, we stratified the studied sample as low daily frequency consumers of milk, fresh DP, and cheese when participants reported consuming 0 time/day for milk, and <0.5 time/day for fresh DP or cheese, respectively. The high frequency was respectively defined for consumptions of >1 time/day of milk, and >1.5 time/day of fresh DP or cheese (Table 1).

#### 3.2.1. Milk

Regarding the socio-demographic characteristics of milk consumers, we observed that the mean age of participants and proportions of women and never smokers were significantly higher with the highest frequency consumption of milk (i.e., 76.7 years for the highest milk intake tertile vs. 75.8 years for the lowest ones, 72.9% women for the highest milk intake tertile vs. 61.2% for the lowest ones, 72.1% never smokers for the highest milk intake tertile vs. 58.1% for the lowest ones) (Table 1).

With regard to the daily frequency of milk consumption, marginal but significantly lower energy intake was observed among non-consumers of milk with 100 kcal/day less than other consumers (Table 2).

Mean intakes of carbohydrates, SFAs (+1.7 g/day between the highest milk intake tertile and the lowest ones), and proteins (+3.3 g/day between the highest milk intake tertile and the lowest ones) from animal sources were significantly higher with the higher frequency of milk consumption, while the total PUFAs, in particular the omega-6 PUFAs, intake was lower with a higher frequency of milk consumption (all *p*-value global < 0.05). The proportion of participants in line with the RDA for proteins significantly increased with the frequency of milk intake. Calcium, phosphorus, and vitamin B2 were the only micronutrients provided by DP whose intakes were higher with the higher frequency of milk consumption. The proportion of participants in line with the RDA for calcium significantly increased with the frequency of milk intake. Moreover, the frequency consumption of milk was not significantly associated with the frequency consumption of cheese, but the higher the frequency of milk consumption, the higher the frequency of fresh DP, biscuit, and sweet intakes (Table 3). On the other hand, a higher frequency of milk intake was significantly associated with a lower frequency intake of charcuterie, meat, coffee, and alcohol. A U-shaped relationship was observed between milk and tea intake (Table 3). The frequency consumption of all other food groups was not significantly associated with that of milk. Finally, the frequency of milk intake was not significantly associated with the amount of cheese consumed but was significantly associated with higher amounts of milk and fresh DP intake (Table 4).

#### 3.2.2. Fresh DP

Second, regarding the frequency consumption of fresh DP, sex, marital status, and income were all significantly associated with fresh DP intake: participants with the highest fresh DP frequency consumption were more often women (72.0% for the highest fresh DP intake tertile vs. 47.0% for the lowest ones), widowed (40.5% for the highest fresh DP intake tertile vs. 27.3% for the lowest ones), and reported the lowest incomes. Among other characteristics, the frequency of consumption of fresh DP was significantly associated with social isolation and smoking status; the moderate consumers being less isolated (+1% of isolated participants with highest fresh DP intakes compared with the lowest ones), and the lowest fresh DP consumers more often being current or ex-smokers than the others (Table 1).

The frequency of fresh DP intake was significantly associated with the reported daily total energy intake of participants; a higher mean energy intake was reported among participants with the lowest frequency consumption of fresh DP. The consumption of total fatty acids, including MUFAs, total PUFAs, and omega-3 PUFAs, proteins from vegetable origins, and fiber were significantly lower among participants with the highest frequency consumption of fresh DP (Table 2). Again, the reported consumptions of calcium, phosphorus, and vitamin B2, in part provided by DP, were the highest when the frequency of fresh DP consumption was the highest. The higher frequency consumption of fresh DP was significantly associated with lower intakes of fiber, iron, and vitamins PP and B12 (Table 2). The proportion of participants in line with the RDA for calcium, but not for protein, significantly increased with the frequency of fresh DP intake.

The frequency consumption of fresh DP was significantly positively associated with that of milk while inversely associated with that of cheese (Table 3). The consumed amount of fresh DP was significantly higher among participants with the highest frequency consumption of milk and lower among participants with the highest frequency consumption of cheese, compared with the lowest frequency consumers (92 g/day vs. 43 g/day on average and 67 g/day vs. 103 g/day on average, respectively) (Table 4). In the study of all food groups recorded in the FFQ database, when the frequency of consumption of fresh DP was highest, the frequency consumption of rice, eggs, and tea was highest, while the frequency consumption of cereals, pulses, charcuterie, meat, and alcohol was lowest. The frequency consumption of all other food groups was not significantly associated with that of fresh DP (Table 3).

#### 3.2.3. Cheese

Third, regarding the frequency of cheese intake, participants with the highest report were significantly more often men (48.2% men for the highest cheese intake tertile vs. 29.1% for the lowest ones) and married (59.9% married men for the highest cheese intake tertile vs. 50.5% for the lowest ones). The frequency consumption of cheese was significantly associated with social isolation and smoking status: participants with the highest frequency of cheese intake were less often isolated (5.5% for the highest cheese intake tertile vs. 10.2% for the lowest ones) and never smokers (58.0% for the highest cheese intake tertile vs. 67.2% for the lowest ones) (Table 1).

The reported daily total energy intake of participants was significantly associated with their cheese intake, as the highest consumers reported 370 kcal/day more than the lowest consumers on average. The consumption of carbohydrates, total and all sub-types fatty acids, proteins (from animal and vegetable sources), fiber, calcium, phosphorus, zinc, and all vitamins provided in part by DP were higher among participants with the highest frequency of cheese consumption (Table 2). The proportion of participants in line with the RDA for proteins and calcium significantly increased with the frequency of cheese intake. The frequency of consumption of cheese was inversely significantly associated with that of milk and fresh DP. This association was only observed regarding the consumed amount of fresh DP (67 g/day vs. 103 g/day on average for the highest vs. the lowest frequency of cheese consumption categories) but not the consumed amount of milk (Table 4).

When the frequency of consumption of cheese was highest, the frequency consumption of cereals, pulses, pasta, potatoes, rice, sweets, charcuterie, meat, poultry, and alcohol was highest. The frequency consumption of all other food groups was not significantly associated with that of cheese (Table 3).

## 4. Discussion

In this large sample of French elderly community dwellers, we observed that DP frequency consumption was associated with several socio-demographic, dietary characteristics, and lifestyle factors, with specificities according to each DP sub-type. Gender and smoking status appeared as key factors both associated with total DP and each DP sub-type intake, while marital status and social isolation were only associated with fresh DP and cheese frequency consumption, in the opposite direction. Overall, it appears from these results that cheese consumers differed from that of milk and fresh DP: a higher cheese frequency consumption was observed among men, married, less isolated, and more often smokers. Regarding dietary data, both food group and nutrient intakes differed according to the DP sub-type consumed. The fresh DP frequency consumers exhibited different dietary patterns than milk or cheese consumers as observed on the frequency consumptions of cereals, pulses, sweets and chocolate, eggs, and tea. As a consequence, these differences were also observed on a majority of nutrients, except for calcium, phosphorus, and vitamin B2, whose consumptions were always significantly higher regardless of the higher frequency of milk, fresh DP, or cheese consumption.

Few studies have characterized DP consumers, particularly among older adults in France [9,41]. In a previous study focusing on elderly people enrolled in a population-based cohort in south-east France and implemented in 2002 (i.e., at the same time as the present dietary survey) [29], DP consumption appeared as a major provider of both SFA and protein (mainly from animal sources) intakes. This was in accordance with results from the present study, while we added that among DP sub-types, the highest frequency consumption of fresh DP was not the main provider of these particular nutrients. Indeed, specific DP dietary patterns were observed here, since higher frequency consumers of fresh DP were also higher frequency consumers of milk, while higher frequency consumers of cheese were the lowest frequency consumers of milk and fresh DP.

Interestingly, from a recent national survey [42], it appeared that among participants aged 55 to 79 years in 2014, only 19% were aware of the French national guidelines, and 64% reported lower estimates than guidelines. The same results were reported earlier in another national sample of French elderly participants, suggesting that the advancement of knowledge, and possibly, as a consequence, of eating habits, may not yet have improved over time [43]. However, being high consumers of total DP or DP sub-types significantly increased the proportion of participants in line with the national total protein and calcium RDA. This would suggest (i) encouraging the consumption of total DP and particularly of milk and cheese, among this vulnerable population, to ensure adequate intake of protein and calcium [6], and (ii) modifying the guidelines about DP among older adults. However, this would also encourage a higher SFA intake, already above the recommendations among this sample as previously reported [36] and which may be not desired [25,26,27]. On the other hand, the various dietary patterns of DP consumers, whatever the sub-type, hence the multiple providers of SFA, complexed the picture further [23,28]. The best way to communicate about these recommendations on total DP, DP sub-types, and protein and calcium intake remains a public health challenge [5]. Indeed, when comparing the present results established on a sample of older people 67 years and over in 2001 with recent ones, we emphasize the secular trend for a decreased consumption of total DP over time in France. However, we also already described that the intake of major food groups appeared relatively stable during a follow-up in 3C-Bordeaux [44]. Despite the traditional French culinary cultural habits, two national surveys (i.e., the INCA2 and INCA3 studies) also reported that skipping breakfast (usually associated with a higher consumption of milk) becomes common, as well the simplification of main meals characterized by a single dish and therefore the absence of dessert, and possibly of yogurt [9,45].

Regarding the dietary patterns of the studied sample, we observed that the other recorded food groups’ intake was distinctive features of each DP sub-type consumer. Briefly, the highest frequency consumers of milk faced a “biscuits and snacking” pattern, already identified among this cohort [35], of mainly women, who we could imagine dipping their biscuits in the milk. For the highest frequency consumers of fresh DP, we would be in the presence of a “low total energy intake” pattern, described as widowed and isolated women with low incomes. This can be compared with the “small eaters” pattern already characterized among this cohort [35]. For the highest frequency consumers of cheese, their overall dietary pattern referred to a “bon vivant” pattern, mainly characterized by men, who we could imagine consuming cheese in a friendly atmosphere, eating a piece of bread, a piece of sausage, drinking wine, and smoking. This last pattern could be compared with the “charcuterie-meat-alcohol” dietary pattern already identified by another statistical approach in this cohort but considering total DP intakes [35]. Here, it appears that “cheese” could be considered as the fourth component of such a dietary pattern, also known as the “traditional pattern” or “western diet” [46], and encourages a split of the total DP food group as separated components to build data-driven dietary patterns. It should be acknowledged that high cheese consumption is a hallmark of French dietary habits. Already, in 2009, Sofi et al. reported that Greece and France were countries from the Mediterranean basin with the highest consumption of cheese [47]. More recently, a report among the SHARE database reported considerable heterogeneity in DP consumption across Europe, with higher levels in central and northern countries and in Spain, and the lowest prevalence of dairy intake in eastern European countries [48]. Finally, the EFSA survey also reported that France and Italy were both countries with a large consumption of cheese, and that France is represented by low consumers of milk [49]. Altogether, the present results were in line with these previous observations.

As expected, several socio-demographic and lifestyle characteristics were associated with the consumption of total DP in the present studied sample, and our data added details on their associations with DP sub-types. Indeed, gender is a largely recognized factor associated with dietary habits, and our results confirmed that men were more likely high-frequency cheese consumers than women, who in turn were more often classified as higher milk and fresh DP frequency consumers in this sample [35,50]. An association between the frequency consumption of cheese and income would have been expected [51]: the maxim ‘there is no good meal without cheese’ appears as a key determinant of the dietary habits of these French participants, whatever the expensive costs of cheese [36]. Decreased perceived attractiveness of food with increased age in terms of taste, appetite, and palatability of food was also commonly admitted [52]. It may encourage elderly persons to choose more tasty cheese in addition to their traditional habits. Finally, smoking status was also differentially associated with the frequency of DP sub-types, as already observed in a previous study reporting that French and worldwide yogurt consumers, more often never smokers, had a better quality diet and lifestyle than non-consumers [53]. Across Europe, gender and age have also been associated with different total DP intakes, with women being greater consumers than men and older adults of 80 years and more being lesser consumers than their younger counterparts [48]. Among environmental factors, the influence of family relations on DP intakes has been reported, such as, for instance, the similarity between mothers and daughters in dairy-related dietary patterns [54]. In the present study, family relations were only assessed by marital status (including married, widowed, or separated and single people). The influence of family relations on DP intakes was illustrated by the fact that men were more often married and cheese consumers, and women more often widowed and fresh DP consumers.

We acknowledge that the accuracy of food intake assessment is crucial in dietary studies, and that performing a single 24-h dietary recall may have induced underestimations of nutrient intakes and intra-individual variations. This methodology also prevented us from assessing the possible loss of vitamins, minerals, and energy between the two surveys. However, a large sample size, even a single dietary survey, may be used to determine the average intake in defined subgroups of a population [55]. Moreover, results from the present study were in line with a previous national report (i.e., INCA 3 study, implemented in 2017 and using a quantitative dietary approach), where the consumed amounts of milk, fresh DP, and cheese were quite similar [42]. Finally, the 24-h dietary recall was administered at the same time as a comprehensive FFQ to collect weekly eating habits, and both surveys exhibited a high concordance between several food groups and nutrient intakes [37,44,56]. Since the present study is cross-sectional and observational, it prevented us from drawing definite conclusions on the associations between DP intakes and socio-demographics, lifestyle, or dietary data and some residual confounding could also explain our observations. The delay of 18 years between the 2 dietary surveys might have decreased the relevance of the present findings, while (i) the French RDA applied in 2001 for older adults is still in progress in 2020, (ii) the DP (and mainly cheese) intakes are part of the hallmark of French dietary habits [47], and it is unlikely that the characteristics of DP consumers have changed dramatically during this period, and (iii) understanding the correlates of DP consumption in year 2001–2002 can still inform today’s DP consumption in the context of the life course approach of nutrition on health. Therefore, collecting this much data appears valuable and can still be informative. Finally, the representative nature of the sample needs to be established before our results can be extended to a larger sample of French elderly as a whole and conclusions drawn with regard to the prevention of inappropriate nutrient intake. Therefore, our results cannot be generalized to populations from different geographic areas with different socio-demographic backgrounds and/or cultural dietary habits. The strengths of the present study included the large sample size, the use of complementary dietary surveys, and the involvement of elderly community dwellers for whom DP recommendations appeared essential to prevent inadequate nutrient intake and possibly disease onset. Finally, this kind of study about non-dietary factors related to total DP and DP sub-type intakes remains strongly limited.

Thanks to the present cross-sectional study, it was possible to identify socio-demographic characteristics and lifestyle factors associated with quantitative and qualitative DP intakes in a French elderly group. It appears that each DP sub-type was also part of distinctive dietary patterns, which encourages individual consideration of these food groups in further analyses on nutrient adequacy among older adults.

## Figures and Tables

**Table 1:** Socio-demographic and lifestyle characteristics across increasing daily frequency consumption of dairy products among elderly community dwellers from the 3C study, Bordeaux (France), 2001–2002, *n* = 1584.

	Total Dairy Products (Time/Day)				Milk (Time/Day)				Fresh Dairy Products (Time/Day)				Cheese (Time/Day)			
	**≤2**	**2–4**	**≥4**		**0**	**0–1**	**>1**		**<0.5**	**0.5–1.5**	**>1.5**		**≤0.5**	**0.5–1.5**	**>1.5**	
	***n* = 394**	***n* = 820**	***n* = 370**	***p***	***n* = 456**	***n* = 766**	***n* = 362**	***p***	***n* = 428**	***n* = 770**	***n* = 386**	***p***	***n* = 317**	***n* = 831**	***n* = 436**	***p***
**Sex, women**	223 (56.6)	507 (61.8)	252 (68.1)	0.005	279 (61.2)	439 (57.3)	264 (72.9)	<0.0001	201 (47.0)	503 (65.3)	278 (72.0)	<0.0001	225 (70.9)	531 (63.9)	226 (51.8)	<0.0001
**Age (years) (m (SD))**	75.7 (4.9)	76.4 (5.0)	76.2 (4.9)	0.08	75.8 (4.7) ^‡^	76.1 (5.0)	76.7 (5.2)	0.03	75.9 (5.0)	76.3 (4.9)	76.3 (5.1)	0.38	75.9 (5.4)	76.3 (4.9)	76.1 (4.7)	0.21
**Education**				0.32				0.28				0.63				0.67
**No/primary**	123 (31.2)	284 (34.7)	119 (32.3)		139 (30.5)	267 (34.9)	120 (33.2)		140 (32.8)	267 (34.7)	119 (30.9)		95 (30.1)	284 (34.2)	147 (33.8)	
**Secondary or High**	191 (48.5)	384 (46.9)	192 (52.0)		223 (48.9)	360 (47.1)	184 (51.0)		207 (48.5)	361 (46.9)	199 (51.7)		163 (51.6)	392 (47.2)	212 (48.7)	
**University**	80 (20.3)	151 (18.4)	58 (15.7)		94 (20.6)	138 (18.0)	57 (15.8)		80 (18.7)	142 (18.4)	67 (17.4)		58 (18.3)	155 (18.6)	76 (17.5)	
**Marital status**				0.43				0.58				<0.0001				0.03
**Married**	222 (56.3)	454 (55.4)	181 (48.9)		247 (54.2)	424 (55.3)	186 (51.4)		261 (61.0)	425 (55.2)	171 (44.3)		160 (50.5)	436 (52.5)	261 (59.9)	
**Divorced/separated**	26 (6.6)	60 (7.3)	34 (9.2)		42 (9.2)	51 (6.7)	27 (7.5)		29 (6.8)	53 (6.9)	38 (9.8)		23 (7.3)	62 (7.4)	35 (8.0)	
**Widowed**	122 (31.0)	254 (31.0)	129 (34.9)		141 (30.9)	242 (31.6)	122 (33.7)		117 (27.3)	232 (30.1)	156 (40.5)		118 (37.2)	275 (33.1)	112 (25.7)	
**Single**	24 (6.1)	52 (6.3)	26 (7.0)		26 (5.7)	49 (6.4)	27 (7.5)		21 (4.9)	60 (7.8)	21 (5.4)		16 (5.0)	58 (7.0)	28 (6.4)	
**Monthly income**				0.25				0.29				<0.0001				0.12
**Very low**	25 (6.3)	56 (6.8)	30 (8.1)		34 (7.4)	49 (6.4)	28 (7.7)		18 (4.2)	63 (8.2)	30 (7.8)		19 (6.0)	61 (7.3)	31 (7.1)	
**Low**	108 (27.4)	245 (29.9)	122 (33.0)		122 (26.8)	232 (30.3)	121 (33.4)		123 (28.7)	216 (28.0)	136 (35.2)		91 (28.7)	261 (31.4)	123 (28.2)	
**Average**	104 (26.4)	211 (25.7)	83 (22.4)		113 (24.8)	207 (27.0)	78 (21.5)		118 (27.6)	204 (26.5)	76 (19.7)		83 (26.2)	199 (24.0)	116 (26.6)	
**High**	124 (31.5)	257 (31.4)	100 (27.0)		147 (32.2)	228 (29.8)	106 (29.3)		142 (33.2)	243 (31.6)	96 (24.9)		89 (28.1)	262 (31.5)	130 (29.8)	
**Refused answer**	33 (8.4)	51 (6.2)	35 (9.5)		40(8.8)	50 (6.5)	29 (8.0)		27 (6.3)	44 (5.7)	48 (12.4)		35 (11.0)	48 (5.8)	36 (8.3)	
**Drugs/day ≥6**	148 (37.6)	308 (37.6)	163 (44.0)	0.08	168 (36.8)	305 (39.8)	146 (40.3)	0.50	153 (35.7)	306 (39.7)	160 (41.4)	0.22	122 (38.5)	322 (38.7)	175 (40.1)	0.86
**Social isolation**	38 (9.7)	57 (7.0)	32 (8.8)	0.25	41 (9.0)	49 (6.5)	37 (10.4)	0.06	42 (9.9)	44 (5.8)	41 (10.8)	0.004	32 (10.2)	71 (8.7)	24 (5.5)	0.049
**Smoking status**				<0.0001				0.0005				<0.0001				0.02
**Never smoker**	209 (53.0)	546 (66.6)	253 (68.4)		265 (58.1)	482 (62.9)	261 (72.1)		219 (51.2)	521 (67.7)	268 (69.4)		213 (67.2)	542 (65.2)	253 (58.0)	
**Ex-smoker**	152 (38.6)	236 (28.8)	105 (28.4)		160 (35.1)	241 (31.5)	92 (25.4)		175 (40.9)	215 (27.9)	103 (26.7)		86 (27.1)	244 (29.4)	163 (37.4)	
**Current smoker**	33 (8.4)	38 (4.6)	12 (3.2)		31 (6.8)	43 (5.6)	9 (2.5)		34 (7.9)	34 (4.4)	15 (3.9)		18 (5.7)	45 (5.4)	20 (4.6)	
**Stoutness ^1^**				0.51				0.98				0.91				0.23
**Thinness**	45 (11.7)	90 (11.3)	45 (12.5)		52 (11.8)	84 (11.3)	44 (12.5)		50 (12.0)	87 (11.5)	43 (11.6)		35 (11.4)	93 (11.5)	52 (12.3)	
**Normal**	174 (45.3)	403 (50.5)	174 (48.5)		213 (48.3)	367 (49.1)	171 (48.4)		198 (47.5)	377 (50.0)	176 (47.6)		135 (43.8)	398 (49.2)	218 (51.4)	
**Overweight/obesity**	165 (43.0)	305 (38.2)	140 (39.0)		176 (39.9)	296 (39.6)	138 (39.1)		169 (40.5)	290 (38.5)	151 (40.8)		138 (44.8)	318 (39.3)	154 (36.3)	
**Physical activity**								0.18				0.19				0.29
**Yes**	103 (26.1)	224 (27.3)	102 (27.6)	0.02	127 (27.9)	196 (25.6)	106 (29.3)		111 (25.9)	214 (27.8)	104 (26.9)		89 (28.1)	221 (26.6)	119 (27.3)	
**No**	235 (59.7)	458 (55.9)	184 (49.7)		251 (55.0)	444 (58.0)	182 (50.3)		256 (59.8)	412 (53.5)	209 (54.2)		174 (54.9)	476 (57.3)	227 (52.1)	
**Missing**	56 (14.2)	138 (16.8)	84 (22.7)		78 (17.1)	126 (16.4)	74 (20.4)		61 (14.3)	144 (18.7)	73 (18.9)		54 (17.0)	134 (16.1)	90 (20.6)	

Values are n (%) except where mentioned ^1^ Stoutness was based on Body Mass Index (kg/m^2^) and on “Global Leadership Initiative on Malnutrition” criteria: thinness (if BMI < 20 and if < 70 years) OR (if BMI < 22 AND if ≥ 70 years)/normal (if BMI (20–27) AND if < 70 years) or (if BMI (22–27) AND if ≥ 70 years)/overweight-obesity if BMI > 27 <1% missing values for social isolation and BMI, (1–5)% missing values for education ^‡^ mean value of low category was significantly different from high category (pairwise comparisons Tukey–Kramer test). BMI, Body Mass Index; SD, standard deviation.

**Table 2:** Daily energy, macro- and micro-nutrient intakes across increasing daily frequency consumption of dairy products among elderly community dwellers from the 3C study, Bordeaux (France), 2001–2002, *n* = 1584.

	Total Dairy Products (Time/Day)				Milk (Time/Day)				Fresh Dairy Products (Time/Day)				Cheese (Time/Day)			
	**≤2**	**2–4**	**≥4**		**0**	**0–1**	**>1**		**<0.5**	**0.5–1.5**	**>1.5**		**≤0.5**	**0.5–1.5**	**>1.5**	
	***n* = 394**	***n* = 820**	***n* = 370**	***p***	***n* = 456**	***n* = 766**	***n* = 362**	*p*	***n* = 428**	***n* = 770**	***n* = 386**	***p***	***n* = 317**	***n* = 831**	***n* = 436**	***p***
**Energy (Kcal)**	1624 (545) ^‡^	1697 (528) ^§^	1830 (549)	<0.0001	1645 (550) ^†^	1745 (539)	1716 (532)	0.007	1755 (536)	1693 (539)	1692 (554)	0.049	1509 (483) ^†,‡^	1698 (535) ^§^	1878 (542)	<0.0001
**Macronutrients**																
**Carbohydrates (g)**	182.9 (70.4) ^†,‡^	193.8 (67.2) ^§^	211.6 (72.4)	<0.0001	183.0 (70.0) ^†,‡^	198.8 (70.1)	203.3 (67.5)	<0.0001	191.5 (69.8)	194.6 (68.1)	200.7 (73.4)	0.18	183.4 (67.0) ^†,‡^	195.1 (69.8)	204.2 (71.2)	0.0001
**Total Fatty Acids (g)**	56.8 (27.8) ^‡^	59.2 (27.2) ^§^	64.6 (28.3)	0.0001	58.2 (27.8)	61.1 (27.5)	59.3 (28.2)	0.11	63.2 (28.3) ^†‡^	58.9 (27.3)	58.2 (27.8)	0.005	48.4 (21.9) ^†,‡^	60.1 (28.3) ^§^	67.7 (27.7)	<0.0001
**SFA (g)**	23.4 (12.8) ^†,‡^	25.4 (12.7) ^§^	29.1 (13.6)	<0.0001	24.6 (13.3)	26.1 (12.6)	26.3 (13.7)	0.03	26.8 (13.2)	25.4 (12.8)	25.2 (13.5)	0.07	19.7 (10.5) ^†,‡^	25.6 (12.9) ^§^	30.4 (13.4)	<0.0001
**MUFA (g)**	20.6 (10.9)	21.3 (11.1)	22.3 (11.1)	0.06	20.8 (11.0)	21.9 (11.2)	20.9 (11.0)	0.16	22.9 (11.0) ^†,‡^	21.0 (11.0)	20.5 (11.2)	0.0002	17.6 (9.0) ^†,‡^	21.6 (11.5) ^§^	23.7 (11.0)	<0.0001
**PUFA (g)**	8.7 (6.7)	8.2 (5.7)	8.6 (5.9)	0.51	8.4 (5.9)	8.7 (6.2)	7.9 (5.8)	0.01	9.1 (6.8) ^†^	8.2 (5.7)	8.3 (5.5)	0.04	7.5 (5.2) ^†,‡^	8.6 (6.4)	8.9 (5.7)	<0.0001
**Omega-3 PUFA (g)**	1.35 (1.68)	1.18 (1.29)	1.26 (1.35)	0.28	1.29 (1.53)	1.27 (1.46)	1.12 (1.11)	0.22	1.42 (1.63) ^†^	1.15 (1.33)	1.21 (1.28)	0.0002	1.07 (1.21)	1.27 (1.53)	1.30 (1.30)	<0.0001
**Omega-6 PUFA (g)**	6.7 (5.8)	6.4 (4.9)	6.6 (5.2)	0.79	6.5 (5.2)	6.7 (5.3)	6.1 (5.1)	0.01	7.0 (5.9)	6.3 (5.0)	6.3 (4.8)	0.13	5.7 (4.7) ^†,‡^	6.6 (5.5)	6.9 (5.0)	<0.0001
**Proteins (g)**	71.2 (26.8) ^‡^	74.6 (26.0) ^§^	81.6 (28.0)	<0.0001	73.3 (28.3)	76.1 (26.3)	76.5 (26.3)	0.053	76.3 (26.3)	74.7 (26.9)	75.8 (27.6)	0.39	67.5 (23.6) ^†,‡^	74.2 (26.6) ^§^	83.4 (27.7)	<0.0001
**≥1 g total protein/kg, n (%)**	183 (46.8)	447 (55.2)	251 (68.6)	<0.0001	231 (51.2)	426 (56.3)	224 (62.4)	0.006	240 (56.6)	418 (54.6)	223 (59.0)	0.37	138 (43.8)	442 (54.0)	301 (69.5)	<0.0001
**Animal sources (g)**	49.9 (23.6) ^‡^	53.1 (23.7) ^§^	59.8 (24.8)	<0.0001	52.1 (25.2)	54.2 (23.8)	55.4 (23.7)	0.04	53.9 (23.2)	53.2 (24.5)	55.0 (24.7)	0.41	48.1 (21.8) ^†,‡^	52.6 (23.9) ^§^	60.4 (25.1)	<0.0001
**Vegetable sources (g)**	21.2 (9.4)	21.5 (8.9)	21.9 (9.2)	0.56	21.2 (9.6)	21.9 (9.0)	21.2 (8.4)	0.13	22.4 (9.3) ^‡^	21.5 (8.9)	20.7 (9.2)	0.02	19.4 (8.0) ^†,‡^	21.6 (9.2) ^§^	23.0 (9.3)	<0.0001
**Micronutrients**																
**Fibers (g)**	17.4 (7.8)	17.4 (7.7)	17.5 (8.2)	0.99	17.5 (8.2)	17.4 (7.6)	17.3 (8.0)	0.90	18.1 (7.6) ^‡^	17.3 (7.4)	16.8 (8.8)	0.009	16.4 (7.5) ^‡^	17.2 (7.7) ^§^	18.5 (8.2)	0.001
**Calcium (mg)**	671 (373) ^†,‡^	854 (397) ^§^	1096 (469)	<0.0001	752 (410) ^†,‡^	868 (419) ^§^	1001 (459)	<0.0001	785 (436) ^†,‡^	859 (432) ^§^	966 (420)	<0.0001	711 (328) ^†,‡^	847 (415) ^§^	1012 (492)	<0.0001
**≥1200 mg Calcium, n (%)**	31 (7.9)	131 (16.0)	122 (33.0)	<0.0001	63 (13.8)	123 (16.1)	98 (27.1)	<0.0001	62 (14.5)	127 (16.5)	95 (24.6)	0.0003	25 (7.9)	136 (16.4)	123 (28.2)	<0.0001
**Iron (mg)**	11.0 (6.1)	11.0 (5.4)	11.3 (5.7)	0.63	11.0 (6.0)	11.1 (5.3)	10.9 (5.7)	0.38	11.7 (6.2) ^‡^	11.0 (5.5)	10.5 (5.2)	0.001	10.1 (4.5) ^‡^	10.9 (5.6) ^§^	12.1 (6.2)	<0.0001
**Phosphorus (mg)**	998 (381) ^†,‡^	1093 (360) ^§^	1272 (412)	<0.0001	1045 (401) ^†,‡^	1115 (369) ^§^	1187 (407)	<0.0001	1089 (382) ^‡^	1101 (391)	1157 (396)	0.03	980 (316) ^†,‡^	1094 (386) ^§^	1241 (410)	<0.0001
**Zinc (mg)**	7.6 (7.0)	7.0 (6.1) ^§^	8.1 (7.5)	0.02	8.4 (7.7)	6.8 (6.0)	7.5 (6.6)	0.07	7.2 (6.4)	7.3 (6.3)	7.8 (7.7)	0.99	6.8 (6.8)	7.4 (6.8)	7.9 (6.4)	<0.0001
**Vit B1 (mg)**	0.97 (0.44) ^‡^	1.00 (0.42) ^§^	1.10 (0.47)	0.0002	0.98 (0.44)	1.04 (0.45)	1.03 (0.43)	0.02	1.03 (0.45)	1.00 (0.42)	1.05 (0.47)	0.23	0.99 (0.43)	1.01 (0.44)	1.06 (0.44)	0.04
**Vit B2 (mg)**	1.36 (0.62) ^†,‡^	1.54 (0.67) ^§^	1.81 (0.82)	<0.0001	1.45 (0.75) ^†,‡^	1.56 (0.66) ^§^	1.71 (0.74)	<0.0001	1.44 (0.69)^†,‡^	1.56 (0.70) ^§^	1.69 (0.74)	<0.0001	1.48 (0.64) ^‡^	1.52 (0.64) ^§^	1.70 (0.86)	<0.0001
**Vit PP (mg)**	14.7 (6.9)	14.4 (6.6)	14.4 (7.3)	0.58	15.0 (7.5)	14.5 (6.5)	13.8 (6.7)	0.06	15.1 (6.8)	14.3 (6.5)	14.1 (7.5)	0.03	13.8 (6.3) ^‡^	14.3 (6.7) ^§^	15.4 (7.4)	0.003
**Vit B5 (mg)**	3.6 (1.6) ^†,‡^	4.1 (1.6) ^§^	4.7 (2.0)	<0.0001	3.7 (1.9) ^†,‡^	4.1 (1.6) ^§^	4.5 (1.8)	<0.0001	3.9 (1.8) ^‡^	4.1 (1.6)	4.3 (1.9)	0.005	3.9 (1.6) ^‡^	4.0 (1.7) ^§^	4.4 (2.0)	0.0002
**Vit B6 (mg)**	1.40 (0.58) ^‡^	1.43 (0.56) ^§^	1.52 (0.62)	0.046	1.42 (0.61)	1.46 (0.58)	1.43 (0.56)	0.33	1.47 (0.59)	1.42 (0.53)	1.45 (0.65)	0.55	1.38 (0.54) ^‡^	1.41 (0.57) ^§^	1.55 (0.61)	<0.0001
**Vit B12 (µg)**	5.5 (9.6)	5.3 (10.4)	6.2 (12.8)	0.03	6.1 (12.9)	5.2 (9.6)	5.6 (10.5)	0.49	5.8 (10.1)	5.7 (10.8)	5.1 (11.7)	0.002	4.9 (9.3) ^‡^	5.2 (9.3) ^§^	6.8 (14.1)	<0.0001
**Vit C (mg)**	75.9 (57.2) ^‡^	83.6 (60.9)	87.6 (63.9)	0.02	78.2 (60.5)	83.2 (59.7)	86.9 (63.4)	0.03	77.6 (60.0)	82.9 (57.2)	87.5 (68.0)	0.04	86.3 (64.6)	81.5 (57.7)	82.0 (63.8)	0.44
**Vit D (µg)**	1.81 (3.31)	1.70 (2.60)	1.73 (2.49)	0.002	1.87 (3.34)	1.69 (2.54)	1.66 (2.40)	0.61	1.95 (3.44)	1.61 (2.32)	1.74 (2.75)	0.35	1.67 (2.58)	1.77 (3.07)	1.72 (2.26)	0.009
**Vit E (mg)**	6.4 (4.9)	6.5 (4.3)	6.5 (4.8)	0.15	6.5 (4.9)	6.7 (4.5)	6.0 (4.2)	0.02	6.5 (4.7)	6.4 (4.2)	6.6 (5.1)	0.99	6.2 (5.0)	6.5 (4.5)	6.5 (4.5)	0.01

Daily intakes are derived from the 24-h dietary recall and expressed as mean (Standard Deviation), except where mentioned Abbreviations: SFA Saturated Fatty Acids, MUFA Monounsaturated Fatty Acids, PUFA Polyunsaturated Fatty Acids, Vit Vitamin Missing values for (1–5)% regarding the consumption of proteins >1 g/d ^†^ mean value of low category was significantly different from middle category (pairwise comparisons Tukey–Kramer test) ^‡^ mean value of low category was significantly different from high category (pairwise comparisons Tukey–Kramer test) ^§^ mean value of middle category was significantly different from high category (pairwise comparisons Tukey–Kramer test).

**Table 3:** Mean weekly food groups’ frequency consumption based on the daily frequency consumption of total dairy products and dairy product subtypes among elderly community dwellers from the 3C study, Bordeaux (France), 2001–2002, *n* = 1584.

	Total Dairy Products (Time/Day)				Milk (Time/Day)				Fresh Dairy Products (Time/Day)				Cheese (Time/Day)			
**Frequency**	**≤2**	**2–4**	**≥4**		**0**	**0–1**	**>1**		**<0.5**	**0.5–1.5**	**>1.5**		**≤0.5**	**0.5–1.5**	**>1.5**	
**(Time/Week)**	***n* = 394**	***n* = 820**	***n* = 370**	***p***	***n* = 456**	***n* = 766**	***n* = 362**	***p***	***n* = 428**	***n* = 770**	***n* = 386**	***p***	***n* = 317**	***n* = 831**	***n* = 436**	***p***
**Milk**	2.0 (3.2) ^†^^,‡^	6.3 (3.9) ^§^	11.2 (6.5)	<0.0001	/	/	/	/	5.6 (5.3) ^†,‡^	6.6 (5.5)	6.6 (5.8)	0.003	6.9 (6.2) ^‡^	6.5 (5.4)	5.7 (5.2)	0.02
**Fresh DP**	3.3 (3.6) ^†,‡^	7.2 (4.5) ^§^	11.0 (5.0)	<0.0001	6.7 (5.6) ^‡^	7.0 (4.9) ^§^	7.9 (5.0)	0.0005	/	/	/	/	8.6 (5.7) ^†,‡^	7.0 (4.6) ^§^	6.1 (5.5)	<0.0001
**Cheese**	5.8 (4.2) ^†,‡^	7.4 (4.2) ^§^	10.5 (4.7)	<0.0001	8.1 (4.9)	7.7 (4.5)	7.3 (4.5)	0.11	8.8 (4.7) ^†,‡^	7.4 (4.1)	7.1 (5.2)	<0.0001	/	/	/	/
**Cereals, bread**	18.2 (5.5)	18.6 (5.2)	18.9 (5.7)	0.18	18.2 (5.7)	18.7 (5.1)	18.8 (5.5)	0.23	18.8 (5.0) ^‡^	18.9 (5.1) ^§^	17.8 (6.2)	0.02	17.5 (6.0) ^†,‡^	18.4 (5.3) ^§^	19.7 (4.8)	<0.0001
**Pulses**	0.6 (0.7)	0.6 (0.6)	0.6 (0.8)	0.92	0.6 (0.6)	0.6 (0.7)	0.6 (0.6)	0.91	0.7 (0.8) ^‡^	0.6 (0.6)	0.5 (0.6)	0.001	0.5 (0.7) ^‡^	0.6 (0.6)	0.7 (0.8)	0.002
**Pasta**	2.0 (1.5)	2.0 (1.4)	2.3 (1.8)	0.17	2.1 (1.5)	2.1 (1.4)	2.2 (1.7)	0.83	2.1 (1.5)	2.0 (1.4)	2.3 (1.8)	0.34	2.0 (1.4) ^‡^	2.0 (1.4) ^§^	2.4 (1.7)	0.004
**Potatoes**	2.4 (1.6)	2.7 (1.7)	2.7 (1.8)	0.07	2.6 (1.7)	2.6 (1.7)	2.7 (1.7)	0.62	2.7 (1.6)	2.6 (1.7)	2.5 (1.7)	0.40	2.4 (1.6) ^‡^	2.6 (1.7) ^§^	2.9 (1.8)	0.0002
**Rice**	1.3 (1.4)	1.3 (1.1)	1.4 (1.3)	0.09	1.4 (1.3)	1.3 (1.2)	1.3 (1.2)	0.83	1.2 (1.2)	1.3 (1.2)	1.4 (1.3)	0.04	1.2 (1.2)	1.3 (1.2)	1.4 (1.3)	0.02
**Biscuits, cakes**	1.7 (3.0) ^‡^	2.2 (3.5) ^§^	2.7 (4.1)	0.0006	1.8 (3.1) ^‡^	2.2 (3.4) ^§^	2.8 (4.2)	0.0003	2.0 (3.0)	2.2 (3.6)	2.4 (3.9)	0.18	2.0 (3.3)	2.3 (3.7)	2.2 (3.4)	0.39
**Sweets, chocolate, soda**	7.8 (6.2) ^‡^	8.7 (6.7) ^§^	10.2 (8.1)	0.0006	7.6 (6.2) ^†,‡^	8.8 (6.8) ^§^	10.4 (8.0)	<0.0001	8.4 (6.2)	9.0 (6.9)	8.9 (7.8)	0.53	7.8 (6.6) ^‡^	9.0 (7.0)	9.3 (7.1)	0.01
**Pizza, sandwich**	0.4 (0.7)	0.4 (0.8)	0.5 (0.9)	0.51	0.4 (0.8)	0.4 (0.7)	0.5 (0.9)	0.56	0.4 (0.6)	0.4 (0.8)	0.5 (1.0)	0.66	0.4 (0.7)	0.4 (0.8)	0.4 (0.7)	0.65
**Raw vegetables, salad**	8.7 (5.0)	9.3 (5.1)	8.9 (5.7)	0.07	9.1 (5.3)	9.1 (5.0)	9.0 (5.6)	0.62	8.9 (5.3)	9.0 (4.9)	9.3 (5.8)	0.70	8.7 (5.5)	9.2 (5.0)	9.1 (5.4)	0.12
**Cooked vegetables**	9.5 (4.3) ^†,‡^	10.3 (4.2)	10.6 (4.6)	0.01	9.8 (4.4)	10.4 (4.3)	10.1 (4.3)	0.09	9.8 (4.4)	10.2 (4.1)	10.5 (4.7)	0.15	10.2 (4.5)	10.0 (4.1)	10.4 (4.6)	0.56
**Fruits**	13.4 (7.1)	13.5 (6.5)	13.5 (7.3)	0.53	13.6 (7.3)	13.4 (6.5)	13.5 (7.0)	0.78	13.4 (6.5)	13.4 (6.9)	13.7 (7.2)	0.89	13.1 (6.6)	13.7 (7.0)	13.4 (6.8)	0.46
**Charcuterie**	1.9 (2.4)	1.6 (2.1)	1.5 (2.3)	0.03	1.9 (2.6) ^‡^	1.7 (2.1) ^§^	1.3 (1.8)	0.01	2.1 (2.5) ^†,‡^	1.6 (2.1) ^§^	1.2 (2.1)	<0.0001	1.4 (2.0) ^‡^	1.5 (2.0) ^§^	2.0 (2.7)	0.0005
**Fish, seafood**	2.9 (1.8)	2.9 (1.7)	2.8 (1.8)	0.86	2.9 (1.9)	2.9 (1.7)	2.7 (1.7)	0.17	2.8 (1.7)	2.9 (1.7)	2.9 (1.9)	0.68	2.9 (1.9)	2.8 (1.7)	2.9 (1.8)	0.74
**Eggs**	1.4 (1.1) ^†^	1.5 (1.1)	1.5 (1.2)	0.01	1.4 (1.0)	1.5 (1.2)	1.5 (1.1)	0.59	1.4 (1.2)	1.5 (1.0)	1.5 (1.1)	0.03	1.5 (1.1)	1.5 (1.0)	1.5 (1.3)	0.79
**Meat**	5.1 (2.7) ^†^	4.6 (2.3)	4.9 (2.5)	0.04	5.0 (2.5) ^‡^	4.8 (2.5)	4.5 (2.3)	0.04	5.4 (2.6) ^†,‡^	4.6 (2.3)	4.6 (2.4)	<0.0001	4.7 (2.6)^‡^	4.6 (2.3) ^§^	5.3 (2.5)	<0.0001
**Poultry**	1.8 (1.2)	1.8 (1.2)	1.9 (1.4)	0.61	1.9 (1.3)	1.7 (1.2)	1.8 (1.3)	0.49	1.7 (1.2)	1.8 (1.3)	1.9 (1.4)	0.44	1.7 (1.3) ^‡^	1.7 (1.2) ^§^	2.0 (1.4)	0.02
**Coffee**	6.9 (5.5) ^†,‡^	5.3 (4.7)	5.0 (5.3)	<0.0001	8.1 (5.6) ^†,‡^	4.8 (4.6)	4.5 (4.5)	<0.0001	5.4 (5.0)	5.7 (4.9)	6.0 (5.6)	0.46	5.3 (5.1)	5.7 (4.9)	5.9 (5.6)	0.33
**Tea**	2.8 (4.6)	2.6 (4.3)	2.9 (4.9)	0.92	3.3 (4.6) ^†^	2.4 (4.4)	2.8 (4.7)	0.0006	2.2 (4.2) ^†,‡^	2.9 (4.6)	3.1 (4.7)	0.002	2.9 (4.7)	2.8 (4.6)	2.5 (4.2)	0.28
**Alcohol**	11.9 (13.6) ^†,‡^	9.6 (10.9)	8.9 (10.6)	0.009	11.4 (13.7) ^‡^	10.6 (11.2) ^§^	6.9 (8.5)	<0.0001	13.4 (14.0) ^†,‡^	9.3 (10.5) ^§^	7.6 (9.9)	<0.0001	8.0 (10.1) ^‡^	8.9 (9.9) ^§^	13.4 (14.6)	<0.0001

^†^ mean value of the low category was significantly different from the middle category (pairwise comparisons Tukey–Kramer test). ^‡^ mean value of the low category was significantly different from the high category (pairwise comparisons Tukey–Kramer test) ^§^ mean value of the middle category was significantly different from the high category (pairwise comparisons Tukey–Kramer test). Values are mean (Standard Deviation) Abbreviations: DP Dairy Products.

**Table 4:** Mean daily dairy product (and subtypes) intakes based on the weekly frequency consumption of total dairy products and dairy product subtypes among elderly community dwellers from the 3C study, Bordeaux (France), 2001–2002, *n* = 1584.

	Total Dairy Products (Time/Day) *				Milk (Time/Day)				Fresh Dairy Products (Time/Day)				Cheese (Time/Day)			
	**≤2**	**2–4**	**≥4**		**0**	**0–1**	**>1**		**<0.5**	**0.5–1.5**	**>1.5**		**≤0.5**	**0.5–1.5**	**>1.5**	
**Daily Intake**	***n* = 394**	***n* = 820**	***n* = 370**	***p***	***n* = 456**	***n* = 766**	***n* = 362**	***p***	***n* = 428**	***n* = 770**	***n* = 386**	***p***	*n* = 317	*n* = 831	*n* = 436	*p*
**Milk (mL)**	43.1 (105.4) ^†^^,‡^	111.5 (145.1) ^§^	186.7 (185.3)	<0.0001	78.8 (107.6) ^†,‡^	126.5 (147.2) ^§^	217.3 (181.3)	<0.0001	97.7 (144.7)	119.3 (162.8)	113.5 (150.8)	0.02	122.4 (160.2)	111.3 (151.4)	105.9 (159.2)	0.10
**Fresh Dairy Products (g)**	43.2 (84.3) ^†,‡^	83.1 (99.0) ^§^	122.6 (110.9)	<0.0001	42.9 (44.9)	80.1 (97.8)	91.9 (104.2)	0.04	15.1 (47.2) ^†,‡^	80.3 (88.4) ^§^	161.3 (116.8)	<0.0001	103.0 (116.9) ^†,‡^	82.5 (96.7) ^§^	67.1 (98.6)	<0.0001
**Cheese (g)**	33.1 (40.6) ^†,‡^	39.3 (39.4) ^§^	52.6 (45.2)	<0.0001	11.4 (13.7)	39.6 (39.3)	41.1 (42.3)	0.83	48.1 (43.9) ^†,‡^	39.3 (40.9)	36.3 (39.6)	<0.0001	10.6 (22.4) ^†,‡^	38.1 (36.7) ^§^	68.3 (44.1)	<0.0001

* French Dairy Products intake recommendations at the date of the dietary surveys (2001) were 3–4 servings of DP/day (whatever the subclass) Daily consumed amounts are derived from the 24-h dietary recall and expressed as mean (Standard Deviation) ^†^ mean value of the low category was significantly different from the middle category (pairwise comparisons Tukey–Kramer test) ^‡^ mean value of the low category was significantly different from the high category (pairwise comparisons Tukey–Kramer test) ^§^ mean value of the middle category was significantly different from the high category (pairwise comparisons Tukey–Kramer test).
